# Social Comparison Information Influences Intentions to Reduce Single-Use Plastic Water Bottle Consumption

**DOI:** 10.3389/fpsyg.2021.612662

**Published:** 2021-09-28

**Authors:** Kathryn Bruchmann, Sarah M. Chue, Keelin Dillon, Jaime K. Lucas, Kayla Neumann, Charlotte Parque

**Affiliations:** ^1^Department of Psychology, Santa Clara University, Santa Clara, CA, United States; ^2^Psychology Division, University of California, Santa Cruz, Santa Cruz, CA, United States

**Keywords:** social comparison, single-use plastic consumption, people-environment studies, sustainable lifestyle choices, environmental identity, self-evaluation

## Abstract

Single-use plastic consumption is at an all-time high and threatens environmental and human health. College campuses in particular serve as a hub for single-use plastics due to their convenience for students on the go. The present research tests whether social comparison information can influence self-perceptions of single-use plastic consumption and motivate behavior change within the college campus environment. In a controlled experiment, we measured college students' existing plastic water bottle usage and gave them false feedback about their behaviors and relative standing to their classmates: participants in comparison conditions learned they were either above or below average in their plastic water bottle sustainability behaviors. Results indicated that (relative to a no-comparison control), being above average at water bottle sustainability led students to be more satisfied with their sustainability efforts. However, either kind of comparison information (i.e., being above *or* below average) led to greater behavioral intentions to reduce single-use plastic water bottle consumption in the future. This study highlights how comparison information can be used to motivate sustainable behavior change with regards to single-use plastics.

## Introduction

Every minute, an estimated one million plastic water bottles are purchased globally, and fewer than half of them are recycled (Laville and Taylor, 2017). Americans in particular are responsible for the purchase of 50 billion plastic water bottles per year (Laville and Taylor, 2017). A particular hub for single-use plastics is university campuses (e.g., Smyth et al., 2010). University cafeterias, with multiple food vendors and thousands of students coming to and from class, are a prime site to utilize the convenience of single-use plastics (Fast et al., 2019). Moreover, research suggests that it is in fact university students (not faculty or staff) that are driving the single-use plastic consumption (Diez et al., 2018). Vanderbilt University calculated that students on campus purchase 430,000 plastic bottles per year, and that a university class will consume 1.7 million bottles over 4 years (Kopstain, 1970). In response to the alarming consumption of single-use plastics and its detrimental impact on people and the environment, many universities have made moves towards eliminating single-use plastics and catalyzing change in students' attitudes and behaviors (Kopstain, 1970; Bullock, 2019). One commonly used sustainability intervention is providing consumers with comparison information about others' sustainability habits (e.g., Schultz et al., 2019). The goal of the present paper is to test how social comparison information can influence university students' attitudes and motivation toward reducing single-use plastic water bottle consumption.

### Motivating Sustainable Behaviors

In recent years, researchers have tested how to motivate sustainable behaviors; one successful strategy for increasing sustainability behaviors is simply making them easier (Thaler and Sunstein, 2008; Benartzi et al., 2017; Varotto and Spagnolli, 2017). For example, one study showed that simply making reusable dinnerware more visible than single-use alternatives in university cafeterias made people more likely to choose the waste-free option (Manuel et al., 2007).

Some researchers argue that even when structural barriers to environmental action are removed, people do not behave sustainably unless they have a strong pro-environmental attitude (e.g., Gifford, 2011) or environmental identity (e.g., Clayton, 2003, 2017). Feeling more connected to the environment (see Nisbet et al., 2008), predicts more pro-environmental behaviors (Hinds and Sparks, 2008; Perrin and Benassi, 2009; Qasim et al., 2019), and even more frequent participation in environmental activism (Schmitt et al., 2019) or volunteer work (Dresner et al., 2015). As such, many researchers argue that the key to promoting sustainable behaviors is to promote a stronger connection to nature or environmental identity within citizens (see Gifford, 2011). Research suggests that it could develop through personal experiences with nature (Prevot et al., 2016), self-efficacy building education (Estrada et al., 2017), and social interaction (Stapleton, 2015).

More generally, a strong influence on self-perceptions and identity is through comparisons with others (e.g., Festinger, 1954; Suls et al., 2002). The present research tests how social comparison information about single-use plastic consumption can influence both self-perceptions of sustainability and motivation for future sustainable behaviors.

### Social Comparison as Motivation

The vast literature on social comparison theory (Festinger, 1954) demonstrates that relative standing with others influences beliefs about the self and can inspire future behaviors (e.g., Kluger and DeNisi, 1996; Lockwood and Kunda, 1997; Mahler et al., 2010; Bruchmann, 2017; Samek et al., 2020). Indeed, in recent years, researchers have turned to social comparison based interventions to encourage more recycling (Schultz, 1999), as well as less water (Schultz et al., 2019), and electricity consumption (Schultz et al., 2015; see Valnoski, 2019 for a review). For example, in one study researchers provided residents of a neighborhood with comparison information about their water usage relative to their community average (i.e., a social norm; Schultz et al., 2019). Residents who were using more water than average reduced their consumption after receiving the comparison information; however, households that were using *less* water than average continued to consume at a desirable low rate. This study suggests that comparisons with better-off and worse-off others might both lead to desirable outcomes; this may be due to the tendency to use social comparison information to self-enhance (e.g., Bruchmann, 2017). People who learn they are doing better than others might be motivated to maintain their positive sense of self (Wills, 1981). In contrast, people who learn they are doing worse than others are more likely to be motivated to repair their self-image through committing to improved future behaviors (Mahler et al., 2010; Samek et al., 2020).

### The Present Research

The present research offers a test of social comparison information on university students' self-perceptions of single-use plastic consumption and the motivation and desire to reduce single-use plastic consumption. Participants were given false feedback about their single-use plastic water bottle sustainability behaviors, and in some cases learned that their sustainability behaviors were better or worse than the average student at their university. Participants rated their perceptions of their own sustainability, their motivation to change future behaviors, and their belief in their ability to change future behaviors. Since people generally have more favorable self-impressions when comparing to downward targets, we predicted that participants who learned they were above average would feel better about themselves than those who learned they were below average or a baseline control. However, because comparing with upward targets is more likely to motivate and inspire improved future performance, we predicted that participants who learned they were below average (vs. control) would have more sustainable behavioral intentions for the future.

## Methods

### Participants and Design

Undergraduates from a mid-sized university (*N* = 181; *M*_*age*_ = 19.19, *SD*_*age*_= 1.03) were recruited to participate in a study about “water bottle sustainability” in exchange for partial course credit. Participants were predominantly female (65.2%) and predominantly white (61.9%). Sample size was determined by making the *a priori* decision to collect data from as many participants as possible across two school terms. Participants were randomly assigned to one of three experimental conditions: above average, below average, or no-comparison control.

### Materials and Procedures

To begin the online study, participants took a bogus Water Bottle Sustainability Quiz created by the researcher team that ostensibly reliably tested a baseline of plastic water bottle sustainability behaviors. Participants read that the quiz was a “validated measure” that was being tested at their university. The quiz included 10 questions with different answer formats, such as yes/no (e.g., “Do you own a reusable water bottle?”), frequencies (e.g., “Across the last 2 months, how many single-use plastic water bottles have you used?”), or response scales (e.g., “how often do you recycle single-use plastic water bottles,” 1= *never*, 7= *always*). The purpose of these varied scale questions was to ensure that participants did not have a good sense of how they were performing so that the bogus feedback would be believable regardless of their current sustainability behaviors.

#### Comparison Feedback

Upon completion of the quiz, all participants saw that their Water Bottle Sustainability Score was a 78 out of 100. This score was pre-tested with a lab group and a research methods class in order to feel like a middling or “average” score. This numeric feedback was accompanied by the text, “Clearly you have a lot of good sustainable water bottle habits, but there is still room to improve!” For participants in the control condition, this was all the information that was displayed. Participants in the comparison conditions also saw the supposed average scores of students on their campus. Participants in the below average comparison condition learned that the average score was an 85 out of 100 (written in green for emphasis), while participants in the above average comparison condition learned that the average score was a 71 out of 100 (written in red). Note that these two comparison targets were an equal distance away from the participants' own scores of 78.

#### Dependent Measures

Next, participants rated their perceptions of and satisfaction with their Water Bottle Sustainability Score (1 = *very poor*/*dissatisfied*, 7= *very good*/*satisfied*). Participants' ratings of and satisfaction with their sustainability quiz scores were averaged to create a self-evaluation composite (α = 0.71). Then, participants indicated their likelihood of trying to reduce single-use plastic water bottle consumption in the future, and how motivated they were to improve their water bottle sustainability (1 = *extremely unlikely*/*unmotivated*, 7 = *extremely likely*/*motivated*). Participants' reported likelihood of and motivation to decrease single-use plastic water bottle consumption were aggregated to form a behavioral intention composite (α = 0.89). In addition, participants also rated if they thought they were able to improve their water bottle sustainability in the future (1 = *definitely no*, 7 = *definitely yes*).

#### Environmental Identity

Next, participants indicated how much they identified with nature and the environment. First, participants responded to an 11-item scale measuring Nature Identity (Prevot et al., 2016; α = 0.87 in the current sample). Participants indicated agreement with statements such as “I feel that I am part of nature, not separate from it” (1 = *strongly disagree*, 5 = *strongly agree*). Second, participants completed the three-item Environmental Self-Identity Scale (van der Werff et al., 2013; α = 0.89 in the current sample) by indicating agreement with statements like “Acting environmentally friendly is an important part of who I am” (1 = *strongly disagree*, 7 = s*trongly agree)*. Finally, participants were asked to recall their own scores and the average scores on the sustainability quiz, were probed for suspicion about the comparison information and the purpose of the study, and were debriefed.

## Results

### Preliminary Analyses

#### Manipulation Check

In order to test whether the social comparison manipulation worked, we examined whether participants were able to recall their own and the average scores. Overall, 98.9% of participants accurately remembered their own score within five points; there was no difference in accuracy across conditions, *F* < 1. Participants in the comparison conditions also overwhelmingly (99.1%) remembered the average comparison information within five points. There was no difference in accuracy between the downward and upward comparison, *t*_(112)_ = 0.90, *p* = 0.371, *d* = 0.09.

#### Pre-existing Water Bottle Sustainability

Analyzing the answers from our Water Bottle Sustainability Quiz allowed us to examine the existing sustainability behaviors of our sample. Nearly all participants (97.2%) reported owning a reusable water bottle, and over half (56.4%) of the participants indicated that they use reusable water bottles “all the time.” Only 18.3% of our sample reported using more than 2 single-use plastic water bottles in a typical week across the previous 2 months. And, nearly all (81.7%) who use single-use plastic water bottles reported disposing of them in the recycling bin. Overall, from this initial survey, we concluded that our population already showed some sustainable behaviors.

#### Environmental Identity

There was not a significant effect of comparison condition on participants' responses to the Nature Identity Scale, *F*_(2, 178)_ = 2.73, *p* = 0.068, η_*p*_^2^ = 0.030, or the Environmental Self-Identity Scale, *F*_(2, 178)_ = 1.54, *p* = 0.217, η_*p*_^2^ = 0.017. One-sample *t*-tests comparing the mean responses to the midpoint of the scale (4) revealed that participants overall felt a strong nature identity, *t*_(180)_ = 14.28, *p* < 0.001, *d* = 1.02, and environmental self-identity, *t*_(180)_ = 10.34, *p* < 0.001, *d* = 1.02. See Table 1 for correlations between nature and environmental identity and our primary dependent measures. Notably, both nature and environmental identity were related to behavioral intentions and ability, and as such, we included them as covariates in our main analyses.

**Table 1:** Means and correlations across dependent measures.

**Dependent measure**	***M* (*SD*)**	**1**	**2**	**3**	**4**	**5**
1. Self-evaluation	4.44 (1.07)	1				
2. Behavioral intention	5.42 (1.28)	−0.12	1			
3. Ability	6.04 (1.21)	0.07	0.38^***^	1		
4. Environmental identity	4.80 (1.04)	−0.15^*^	0.42^***^	0.22^**^	1	
5. Nature identity	5.08 (1.02)	−0.14	0.37^***^	0.24^***^	0.61^***^	1

* *Significance at p = 0.05*,

** *p = 0.01, and*

*** *p = 0.001*.

### Primary Analyses

**Figure 1:**
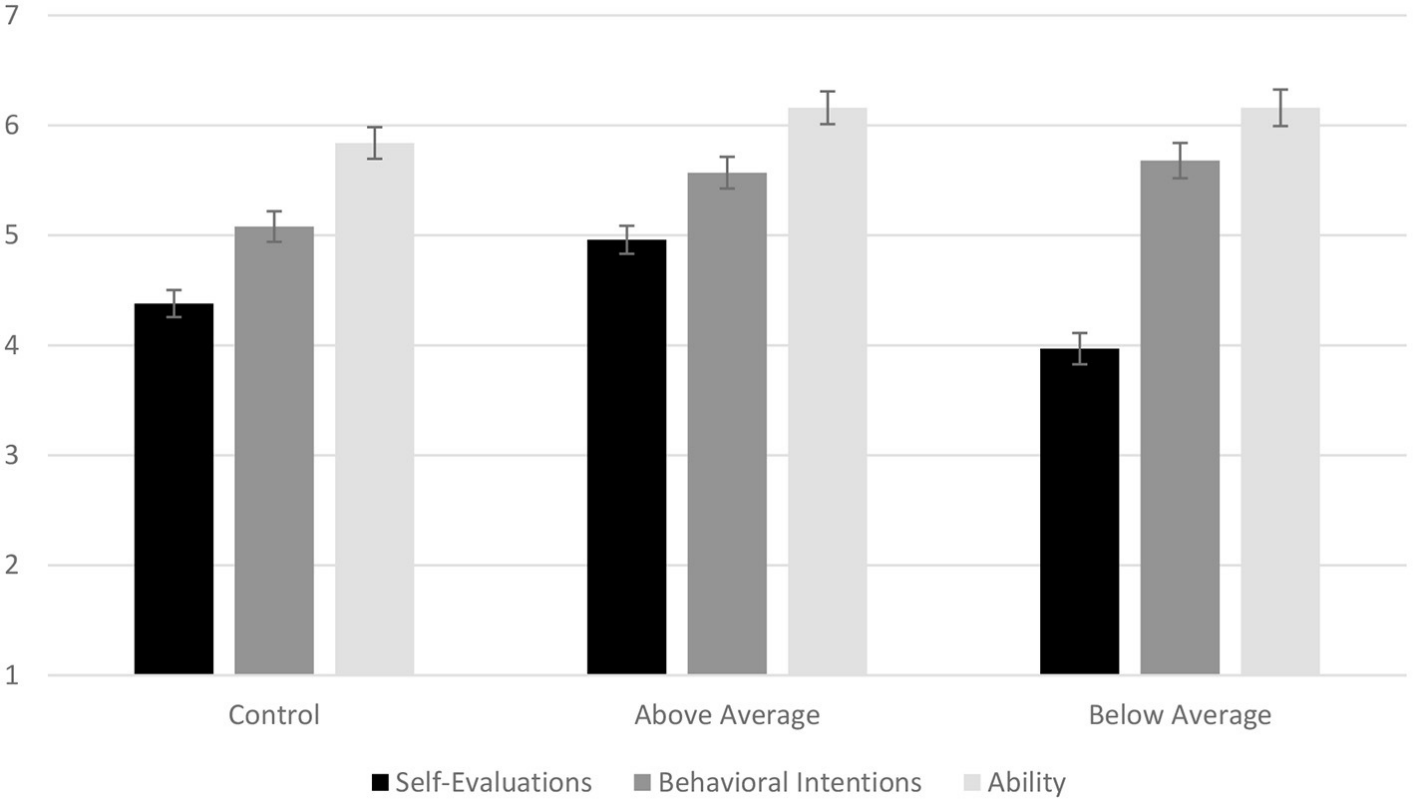
Mean self-evaluations, behavioral intentions, and ability ratings across comparison conditions. Error bars reflect standard errors. Y-axis represents 1–7 response scales.

We conducted a series of one-way ANCOVAs with comparison condition as the independent variable and environmental and nature identity as covariates. Note, patterns for all DVs were similar when covariates were not included. See Figure 1.

#### Self-Evaluations

A significant effect of comparison condition emerged on self-evaluation, *F*_(2, 176)_ = 12.75, *p* < 0.001, η_*p*_^2^= 0.127. As predicted, participants who were told they were above average (*M* = 4.96, *SD* = 0.83) rated themselves more favorably than participants in the baseline control condition (*M* = 4.38, *SD* = 1.14; *p* < 0.001, *d* = 0.58) and participants who were told they were below average (*M* = 3.97, *SD* = 1.02, *p* < 0.001, *d* = 1.06). Additionally, participants who were below average rated themselves less favorably than those in the baseline control condition (*p* = 0.046, *d* = 0.38). Neither of the covariates were related to self-evaluations.

#### Behavioral Intentions

There was a significant effect of comparison condition on behavioral intentions to reduce plastic consumption, *F*_(2, 176)_ = 4.13, *p* = 0.018, η_*p*_^2^ = 0.045. Consistent with our predictions, participants who were told they were below average (*M* = 5.68, *SD* = 1.25) reported stronger behavioral intentions than the baseline control condition (*M* = 5.09, *SD* = 1.35, *p* = 0.046, *d* = 0.45). Contrary to our predictions, participants who were told they were above average (*M* = 5.57, *SD* = 1.78) also reported greater behavioral intentions of future water bottle sustainability behaviors than those in the baseline control condition (*p* = 0.007, *d* = 0.30). There was no difference between participants who were told they were above or below average (*p* = 0.578, *d* = 0.07). There was a significant effect of both covariates: environmental identity, *F*_(1, 176)_ = 12.13, *p* < 0.001, η_*p*_^2^ = 0.064, and nature identity, *F*_(1, 176)_ = 6.06, *p* = 0.015, η_*p*_^2^ = 0.033. Consistent with previous research, this suggests that greater feelings of connection to the environment or nature predict motivation to be more sustainable. Notably, our sample overall reported relatively strong (*M* = 5.42, *SD* = 1.28) behavioral intentions to decrease plastic consumption compared to the midpoint of the scale (4), *t*_(180)_ = 14.92, *p* < 0.001, *d* = 1.28.

#### Perceived Ability

We did not find an effect of comparison condition on participants' perceived ability to decrease single-use plastic water bottle consumption, *F*_(2, 176)_ = 1.76, *p* = 0.190, η_*p*_^2^ = 0.019. A one-sample *t*-test revealed that in general participants rated their ability to become more sustainable (*M* = 6.04, *SD* = 1.21) above the midpoint of the scale (4), *t*_(180)_ = 22.62, *p* < 0.001, *d* = 1.21. There was also a marginal effect of the covariate nature identity, *F*_(1, 176)_ = 3.80, *p* = 0.053, η_*p*_^2^ = 0.02. Consistent with previous research, this suggests that a stronger feeling of connection to nature may be related to self-efficacy surrounding sustainability.

## Discussion

In general, we found evidence that social comparison feedback can be an effective way to motivate the reduction of single-use plastic water bottle consumption. We saw evidence that undergraduates who learned they were above-average in water bottle sustainability behaviors at their university had more favorable self-impressions surrounding their sustainability than a baseline, consistent with research that suggests people use comparison information from worse-off others to self-enhance (Wills, 1981; Bruchmann, 2017).

However, we saw evidence that whether participants learned they were above *or* below average, they had greater intentions to be more sustainable in the future (relative to baseline). As such, it seems likely that different mechanisms account for a motivation to improve for people with upward vs. downward comparison information. For example, learning that their sustainability efforts are below average may be threatening to self-perceptions and lead people to want to repair their self-image. Indeed, research suggests that after threatening feedback, people try to repair self-esteem by self-enhancing (e.g., Friend and Gilbert, 1973) or by reporting higher expectations of future successes (Aspinwall and Taylor, 1993; see Johnson, 2012 for a review). However, learning their sustainability efforts are above average may lead people to want to *maintain* a positive self-image; the self-evaluation maintenance model (Tesser, 1988) suggests that people are motivated to preserve and protect a positive self-image. Thus, expecting that future behaviors will be even more sustainable may be a way to maintain a favorable and sustainable self-image. Additionally, social comparison research suggests that comparisons with better-off others can under certain circumstances lead to feelings of inspiration (e.g., Lockwood and Kunda, 1997) or motivation to improve in the future (e.g., Suls et al., 2002). Assimilation toward better-off others is particularly likely when the comparison targets are relevant and the successes seem attainable (Lockwood and Kunda, 1997); in the case of our study, students were comparing themselves to peers, and generally reported feeling able to improve sustainability behaviors suggesting that they thought other successes were attainable.

Interestingly, we did not see any effect of social comparison information on people's perceived ability to practice more plastic water bottle sustainability. In fact, participants reported that they were very able to improve. This suggests that social comparison information is influencing a *desire* to be more sustainable, and not necessarily concerning self-efficacy beliefs about being sustainable or action plans to achieve their goals.

## Limitations

Because our data is cross-sectional, we were only able to measure behavioral intentions to increase plastic water bottle sustainability behaviors, and were not able to measure actual change in behavior. However, other research suggests that social comparison information from better-off others can and does influence actual behaviors surrounding energy consumption (Schultz et al., 2015) or water usage (Schultz et al., 2019). So, it is possible (or even likely) that students who found out they were below average in plastic water bottle sustainability on their university campus could actually show a change. Whether participants who thought they were above average would actually change behaviors is an empirical question; it is possible that reporting greater behavioral intentions than the baseline condition was a means of self-enhancing and that actual behaviors would not change, especially if other situational barriers emerged (e.g., Kaiser and Schultz, 2009). Consistent with this, in a study about social comparison and skin cancer prevention, Mahler et al. (2010) found that comparisons with worse-off others actually negated positive effects of other interventions and did not increase sunscreen usage among participants. As such, it is important for future research to test whether increased motivation to reduce plastic consumption translates to actual behaviors for social comparison conditions.

Additionally, our sample, which is drawn from a university that boasts several sustainability initiatives (Plan, 2019) and regularly finds itself on lists of “most sustainable college campuses” (e.g., Top 50 Green Colleges, 2020) may not be representative of undergraduates more generally, or a broader population. Because the campus culture overall highly values and emphasizes sustainability, it is likely that students in general feel a stronger environmental identity or more efficacy around sustainability behaviors. This could influence how social comparison information is used; future research should test the effects of social comparison information on single-use plastic water bottle consumption across more diverse populations. Furthermore, future research should recruit higher-powered samples in order to test the generalizability of these preliminary findings.

Finally, our experiment required participants to take a “sustainability quiz” before getting social comparison feedback. In a more naturalistic setting, it is likely that only sustainability inclined participants would be motivated to participate in this type of quiz. Alternately, it is possible that completing the quiz and reflecting on personal single-use plastic consumption before receiving comparison information contributed to the effects. Future research should examine alternative ways to provide social comparison feedback; for example, in campus cafeterias, signs could be displayed near coolers of single-use plastic water bottles that provide comparative information about sustainability-related behaviors across campus.

## Conclusion

This research provides evidence that social comparison information can be used as a way to motivate more sustainable single-use plastic behaviors. And, because of the high plastic usage on university campuses, targeting an undergraduate population to become more sustainable can influence the overall campus culture. As single-use plastic consumption continues to threaten our environment and well-being, motivating even the smallest behavioral changes can have an immense positive impact for current and future generations to come.

## Data Availability Statement

The raw data supporting the conclusions of this article will be made available by the authors, without undue reservation.

## Ethics Statement

The studies involving human participants were reviewed and approved by Santa Clara University Human Subjects Committee. The patients/participants provided their written informed consent to participate in this study.

## Author Contributions

All authors contributed equally and are listed in alphabetical order. KB, SC, KD, KN, and CP contributed to the conception and design of the study. KB, SC, and KN performed the statistical analysis and wrote sections of the manuscript. SC, KD, JL, KN, and CP wrote the first draft of the manuscript. All authors contributed to the manuscript revision, all authors read and approved the submitted version.

## Conflict of Interest

The authors declare that the research was conducted in the absence of any commercial or financial relationships that could be construed as a potential conflict of interest.

## Publisher's Note

All claims expressed in this article are solely those of the authors and do not necessarily represent those of their affiliated organizations, or those of the publisher, the editors and the reviewers. Any product that may be evaluated in this article, or claim that may be made by its manufacturer, is not guaranteed or endorsed by the publisher.
